# When diets fall short: link between unsuccessful weight loss and reduced BDNF levels

**DOI:** 10.3389/fnut.2025.1618927

**Published:** 2025-07-08

**Authors:** Gang Wu, Guifeng Shi, Yafei Ye, Xiaoqin He, Yahong Chen, Cuimin Liu, Meixian Zhang

**Affiliations:** ^1^Department of Pharmacy, Taizhou Hospital of Zhejiang Province Affiliated to Wenzhou Medical University, Linhai, Zhejiang, China; ^2^Department of Preventive Health Care, Taizhou Hospital of Zhejiang Province Affiliated to Wenzhou Medical University, Linhai, Zhejiang, China; ^3^Health Management Center, Taizhou Hospital of Zhejiang Province Affiliated to Wenzhou Medical University, Linhai, Zhejiang, China; ^4^Department of Clinical Nutrition, Taizhou Hospital of Zhejiang Province Affiliated to Wenzhou Medical University, Linhai, Zhejiang, China; ^5^Evidence-Based Medicine Center, Taizhou Hospital of Zhejiang Province Affiliated to Wenzhou Medical University, Linhai, Zhejiang, China

**Keywords:** energy restriction diet, brain-derived neurotrophic factor (BDNF), obesity in men and women, overweight, weight loss, serum

## Abstract

**Objectives:**

Brain-derived neurotrophic factor (BDNF) is a protein essential for brain health and nutrient energy metabolism. This study aims to examine the relationship between blood BDNF levels and obesity and to assess the effect of energy-restricted diets on BDNF levels.

**Method:**

We enrolled 233 individuals with normal weight (*n* = 102), overweight (*n* = 52), and obesity (*n* = 69) and measured their serum BDNF levels. Totally 49 overweight and obese participants then followed a 4-week energy-restricted diet. Paired tests were used to statistically evaluate changes in serum BDNF levels before and after the diet. Based on the effectiveness of weight loss, participants were divided into high- and low-response groups. Changes in BDNF levels before and after the diet were further analyzed separately in the high- and low-response groups for both men and women.

**Results:**

Our findings showed that serum BDNF levels were significantly elevated in overweight and obese adults in the Chinese population studied (*P* = 0.002). Energy restriction led to a significant decrease in BDNF levels in overweight and obese participants (before: 19,605.13 ± 5,706.07 pg/ml, after: 16,437.39 ± 5,365.13 pg/ml, *P* < 0.001). In subgroup analysis, a reduction in BDNF levels was observed only in the female hyporesponsive group (*P* = 0.001).

**Conclusion:**

Serum BDNF levels were elevated in overweight and obese adults in the Chinese population, and an energy-restricted diet reduced serum BDNF. In women, failure to achieve weight loss with an energy-restricted diet may be associated with decreased BDNF levels. Maintaining stable BDNF levels, such as through exercise, should be considered to enhance weight loss outcomes. Confounding factors such as the menstrual cycle, menopausal status, and levels of physical activity should be taken into consideration in future studies.

## 1 Introduction

Obesity significantly increases the risk of various health conditions, including heart disease, diabetes, and certain cancers (1). It has reached epidemic proportions globally, affecting over 650 million adults and resulting in substantial increases in healthcare costs and lost productivity (2). This condition contributes significantly to the burden of chronic diseases, thereby straining public health systems and economies worldwide (3).

Energy restriction has been shown to be effective in reducing body weight (BW) and improving metabolic health in individuals with obesity (4). This dietary intervention can lead to significant reductions in fat mass, insulin resistance, and inflammation, thereby mitigating the adverse health effects associated with obesity (3).

Brain-derived neurotrophic factor (BDNF) is a protein primarily synthesized and secreted by brain regions such as the hypothalamus, hippocampus, and prefrontal cortex, with hypothalamic BDNF being critical associated with energy homeostasis, food intake and BW (5, 6). BDNF plays a crucial role in brain health and cognitive function, and it regulates appetite, glucose, and lipid metabolism (7, 8). Peripheral BDNF, secreted by various sources such as platelets, skeletal muscle, and endothelial cells, plays a significant role in muscle metabolism and vascular protection (9, 10). Substantial evidence suggests that BDNF signaling is associated with energy imbalance and severe obesity in both humans and rodents (11, 12).

Exercise, physical training, and dietary interventions have been shown to significantly influence the levels of BDNF (13). A meta-analysis found that physical exercise is particularly effective in promoting increases in BDNF levels among healthy aged subjects (14). A study found that a low-fat diet could impair cognitive function and reduce BDNF expression (15), whereas another study showed that dietary restriction alone did not have a significant impact (16). A study comparing the effects of 12 weeks exercise and diet on BDNF concentrations in overweight and obese individuals found that while exercise alone reduced BDNF levels in men, diet alone or combined with exercise significantly reduced BDNF levels in women, highlighting the importance of considering sex differences in BDNF response to lifestyle interventions (17).

Nonetheless, the relationship between blood BDNF levels and obesity remains debated, and the effects of energy-restricted diet on BDNF are not fully understood (18). We propose that energy restriction influences BDNF levels, which in turn contributes to weight reduction, and the success of weight loss through an energy-restricted diet is associated with corresponding changes in BDNF.

## 2 Materials and methods

### 2.1 Participants

To clarify the relationship between blood BDNF levels and obesity, participants were recruited at the health management center of Taizhou Hospital of Zhejiang Province in China, from 1 January to 10 March 2020. Body mass index (BMI) values were calculated based on weight and height. Participants with underweight (BMI < 18.5 kg/m^2^) were excluded, and were classified as normal weight (18.5 kg/m^2^ ≤ BMI < 24 kg/m^2^), overweight (24 kg/m^2^ ≤ BMI < 28 kg/m^2^), and obese (BMI ≥ 28 kg/m^2^) according to BMI values(19). Blood pressure was measured on the right upper arm using an electronic sphygmomanometer (Omron, HBP-9021) at rest in a sitting position. The blood was collected at fasting conditions. Serum was collected by centrifuge at 1200 g for 10 min after 3 h clotting at room temperature. Serum total cholesterol (TC), low-density lipoprotein cholesterol (LDL-C), high-density lipoprotein cholesterol (HDL-C), triglyceride (TG), and fasting plasma glucose (FPG) were biochemically measured. Serum BDNF levels were measured by ELISA (Abbexa, Catalog No. abx570037). All laboratory equipment was calibrated. All measures were conducted by a technician who was blind to the assigned intervention.

### 2.2 Energy restriction intervention

To investigate the effect of energy restriction on BDNF levels, participants voluntarily participated in a 4-week energy-restricted diet from 1 November 2021, to 30 November 2022. The inclusion criteria were: ➀ participants aged 18–60 years; ➁ a stable weight change (±4 kg) over the last 3 months; and ➂ meeting at least one of the following criteria: overweight with a BMI ≥ 24 kg/m^2^, fat mass percentage ≥30% for women and ≥25% for men, or waist circumference ≥85 cm for women and ≥90 cm for men. The exclusion criteria were as follows: declined to participate in the 4-week energy-restricted diet; ➀ pregnancy; ➁ blood specimens were not collected before or after energy restriction; ➂ no residual samples of blood specimens for serum analyze; and ➃ lost to follow-up, making it impossible to obtain post-energy restriction diet weight data.

All participants were instructed to adhere to a personalized diet plan, following a 5:2 intermittent fasting regimen for 4 weeks. On fasting days, they replaced three meals per day with a meal replacement (∼800 kcal), while on feeding days, they followed a calorie-restricted diet (1,200–1,400 kcal/day for men and 1,000–1,200 kcal/day for women) at home. All participants were provided with meals and each serving of the meal replacement contained 47 g of nutritional powder (858 kJ), including 12.3 g of protein, 26.1 g of carbohydrates, and 5.2 g of fat. Participants were allowed to supplement their diet with probiotics, dietary fiber, and low glycemic index cookies, and consume 150–200 g of non-starchy, sugar-free vegetables, such as cucumber and tomato, between meals. All participants were required to check in via the WeChat group after finishing each meal. They were also encouraged to drink plenty of water on fasting days. Additionally, all participants engaged in appropriate exercise.

BW, height (Ht), percent body fat (PBF), abdominal circumference (AC), body fat mass (BFM), waist-to-hip ratio (WHR), visceral fat area (VFA), systolic blood pressure (SBP), diastolic blood pressure (DBP), pulse rate (PR), TG, TC, HDL-C, LDL-C, FPG, and BDNF were measured before the start of the energy restriction and after 4 weeks. Participants were classified as high responders if they lost 5% or more of their BW after 4 weeks of intervention; otherwise, they were classified as low responders.

### 2.3 Statistical analysis

Primary outcome of the trial was BW in kg after 4 weeks of intervention. We used G*Power 3.1 software to calculate the sample size. We estimated that an enrollment target of 44 participants would provide the study with greater than 85% statistical power to detect an effect size of 0.3 or more difference in the changes of weight before and after the intervention at a significance level of 0.05 using a two tailed test. Taking into account a about 15% loss to follow-up, the sample size was finally set at 51.

Continuous variables were expressed as mean ± standard deviation if normally distributed, or as median (1st quartile – 3rd quartile) if not. The Kolmogorov–Smirnov test was used to evaluate the normality of the distribution of the variables. Normally distributed data were compared using the Student’s *t*-test for two groups, analysis of variance (ANOVA) for three groups, or paired *t*-test for analysis before and after the intervention. Non-normally distributed data were analyzed using the Mann–Whitney *U* test. Categorical variables were expressed as number (%) and compared using the χ^2^ test or Fisher’s exact test.

## 3 Results

### 3.1 Baseline of the participants

We conducted biochemical tests on 223 participants, comprising 102 normal-weight, 52 overweight, and 69 obese individuals. In terms of demographic data, there were no differences in age and height among the groups, but there were significant differences in gender. The proportion of men in the obese group (68.1%) was significantly higher than in the normal-weight (26.5%) and overweight groups (30.8%) (*P* < 0.001). Significant differences were observed in BW, BMI, SBP, DBP, TG, and HDL-C among the three groups (*P* < 0.001). TC and LDL-C were also significantly different among the three groups at *P* < 0.05. However, FPG did not differ significantly among the three groups (Table 1).

**TABLE 1:** Characteristics of the study population at baseline (*n* = 223).

Variables	All (*n* = 223)	Normal weight (*n* = 102, 45.74%)	Overweight (*n* = 52, 23.32%)	Obesity (*n* = 69, 30.94%)	*P*
Male [*n* (%)]	90 (40.4)	27 (26.5)	16 (30.8)	47 (68.1)	<0.001
Age (years)	37.5 ± 10.9	36.3 ± 9.5	38.2 ± 10.7	38.7 ± 12.8	0.341
Ht (cm)	164.2 ± 8.5	162.7 ± 7.5	163.2 ± 8.8	167.3 ± 8.9	0.806
BW (kg)	72.5 ± 17.5	58.6 ± 7.4	74.2 ± 9.2	91.9 ± 13.7	**<0**.**001**
BMI (kg/m^2^)	26.7 ± 5.0	22.1 ± 1.8	27.8 ± 1.4	32.7 ± 2.5	**<0**.**001**
SBP (mmHg)	125 ± 16	118 ± 13	130 ± 18	133 ± 14	**<0**.**001**
DBP (mmHg)	75 ± 13	69 ± 9	78 ± 14	81 ± 13	**<0**.**001**
TG (mmol/L)	1.40 (0.89–2.03)	1.01 (0.78–1.34)	1.63 (1.01–2.44)	2.02 (1.42–3.13)	**<0**.**001**
TC (mmol/L)	4.87 ± 0.92	4.65 ± 0.93	5.10 ± 0.93	5.00 ± 0.82	**0**.**020**
HDL-C (mmol/L)	1.36 ± 0.32	1.51 ± 0.33	1.30 ± 0.24	1.18 ± 0.26	**<0**.**001**
LDL-C (mmol/L)	2.57 ± 0.68	2.38 ± 0.66	2.74 ± 0.75	2.68 ± 0.59	**0**.**011**
FPG (mmol/L)	5.46 ± 1.60	5.20 ± 0.55	5.50 ± 1.97	5.81 ± 2.15	0.335
BDNF (pg/ml)	18,013.3 ± 4,965.7	16,583.2 ± 4,698.1	19,273.7 ± 4,978.4	19,177.5 ± 4,844.9	**0**.**002**

*P* indicates the significance analyzed by analysis of variance or Mann–Whitney *U* test. Bold indicates *P* values <0.05. Ht, height; BW, body weight; BMI, body mass index; SBP, systolic blood pressure; DBP, diastolic blood pressure; TG, triglycerides; TC, total cholesterol; HDL-C, high-density lipoprotein cholesterol; LDL-C, low-density lipoprotein cholesterol; FPG, fasting plasma glucose; BDNF, brain-derived neurotrophic factor.

### 3.2 BDNF levels in normal weight, overweight, and obese groups

Serum BDNF concentrations were observed to be 16,583.2 ± 4,698.1 pg/ml in the normal weight group, 19,273.7 ± 4,978.4 pg/ml in the overweight group, and 19,177.5 ± 4,844.9 pg/ml in the obese group. Significant differences were noted among the three groups (*P* = 0.002) (see Table 1).

**FIGURE 1:**
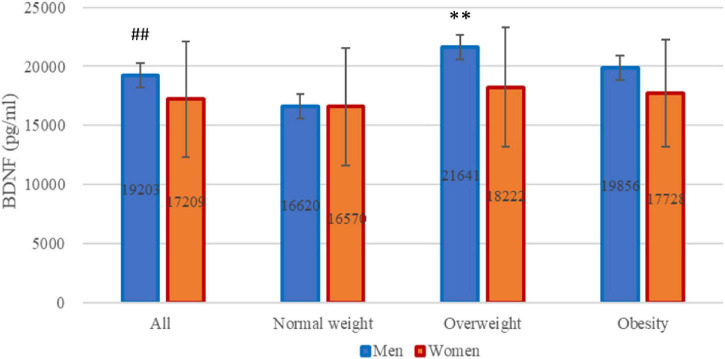
Brain-derived neurotrophic factor (BDNF) levels across all participants. BDNF levels stratified by gender within each weight category: normal weight, overweight, and obesity. ^##^*P* = 0.003 for difference between men and women analyzed by Student’s *t*-test. **P = 0.001 for differences among three weight groups only in men analyzed by analysis of variance.

Given variations in gender distribution across the groups, we further scrutinized BDNF serum concentrations in men and women separately. Levels of BDNF were significantly higher in men than in women (*n* = 90,133 for men and women, respectively, 19,202.6 ± 4,803.8 pg/ml vs. 17,208.5 ± 4,929.0 pg/ml, *P* = 0.003). Intriguingly, distinctions were discernible solely among men (*n* = 27, 16, 47, for normal weight, overweight, and obesity, respectively, *P* = 0.001) with no significant variance observed among women (see Figure 1).

### 3.3 Changes after energy restriction intervention

**FIGURE 2:**
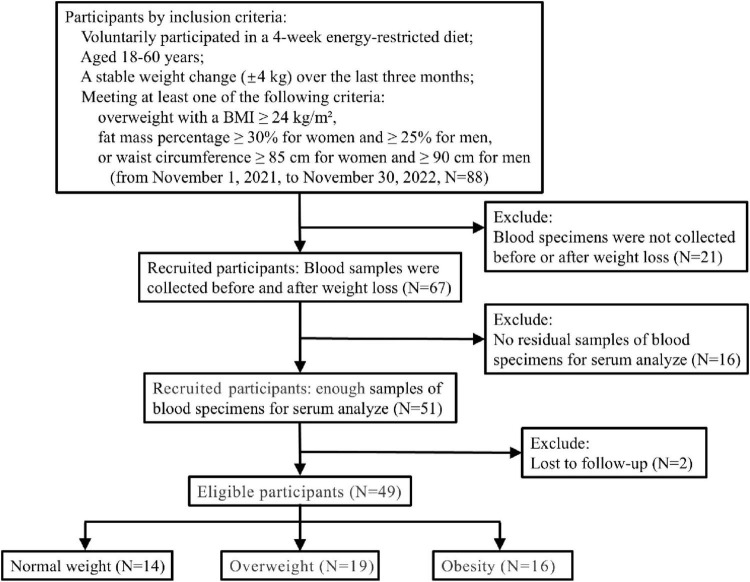
The flowchart for energy restriction intervention.

In total, two participants were lost to follow-up, and 49 individuals took part in an energy restriction intervention study involving an energy-restricted diet from 1 November 2021, to 30 November 2022, when the last participant ended 4-week energy-restricted diet (see the flowchart Figure 2). Remarkably, participants experienced significant reductions in BW, BMI, PBF, AC, WHR, VFA, SBP, PR, and TC levels (*P* < 0.001). Additionally, FPG, TG, and LDL-C showed significant decrease (*P* = 0.004), while DBP exhibited a notable reduction at *P* < 0.05. However, no significant difference was observed in HDL-C levels. Furthermore, serum BDNF levels also exhibited a significant decrease, declining from 19,605.13 ± 5,706.07 pg/ml at baseline to 16,437.39 ± 5,365.13 pg/ml post-intervention (*P* < 0.001). The BDNF in women declining from 17,748.45 ± 5,617.09 pg/ml to 15,157.28 ± 5,082.28 pg/ml (*P* = 0.001), and the BDNF in men declining from 23,100.07 ± 4,083.31 pg/ml to 15,157.28 ± 5,082.28 pg/ml (*P* = 0.003) (see Table 2). The participants experienced no harm other than feeling hungry.

**TABLE 2:** Baseline values and changes in adiposity parameters and cardiometabolic indices (mean ± SD) after 4 weeks diet intervention (*n* = 49).

Variables	Baseline	After 4 weeks	Reduce from baseline	*P*
BW (kg)	75.7 ± 13.9	71.5 ± 13.1	4.2 ± 2.1	**<0**.**001**
BMI (kg/m^2^)	27.9 ± 3.9	26.3 ± 3.7	1.5 ± 0.7	**<0**.**001**
PBF (%)	36.4 ± 6.1	33.5 ± 6.5	2.9 ± 1.5	**<0**.**001**
AC (cm)	96.5 ± 10.3	90.9 ± 9.4	5.6 ± 2.5	**<0**.**001**
WHR	0.95 ± 0.05	0.92 ± 0.05	0.04 ± 0.02	**<0**.**001**
VFA (cm^2^)	133.5 ± 36.2	111.7 ± 34.4	21.9 ± 8.5	**<0**.**001**
SBP (mmHg)	128 ± 20	120 ± 18	8 ± 13	**<0**.**001**
DBP (mmHg)	77 ± 17	74 ± 13	3 ± 10	**0**.**038**
PR (beat/min)	80 ± 10	74 ± 10	7 ± 9	**<0**.**001**
FPG (mmol/L)	5.30 ± 1.16	4.95 ± 0.88	0.35 ± 0.81	**0**.**004**
TG (mmol/L)	1.95 ± 2.06	1.20 ± 0.88	0.75 ± 1.72	**0**.**004**
TC (mmol/L)	4.87 ± 0.92	4.47 ± 0.93	0.40 ± 0.62	**<0**.**001**
LDL-C (mmol/L)	2.46 ± 0.64	2.29 ± 0.61	0.17 ± 0.4	**0**.**004**
HDL-C (mmol/L)	1.32 ± 0.27	1.31 ± 0.25	0.01 ± 0.16	0.578
BDNF (pg/ml)	19,605.13 ± 5,706.07	16,437.39 ± 5,365.13	3,167.74 ± 4,458.93	**<0**.**001**
BDNF in women	17,748.45 ± 5,617.09	15,157.28 ± 5,082.28	2,591.17 ± 4,114.64	**0**.**001**
BDNF in men	23,100.07 ± 4,083.31	18,846.99 ± 8,182.91	4,283.07 ± 4,992.74	**0**.**003**

*P* indicates the significance analyzed by paired *t*-test. Bold indicates *P* values <0.05. BW, body weight; BMI, body mass index; PBF, percent body fat; AC, abdomen circumference; WHR, waist-to-hip ration; VFA, visceral fat area; SBP, systolic blood pressure; DBP, diastolic blood pressure; PR, pulse rate; FPG, fasting plasma glucose; TG, triglycerides; TC, total cholesterol; LDL-C, low-density lipoprotein cholesterol; HDL-C, high-density lipoprotein cholesterol; BDNF, brain-derived neurotrophic factor.

### 3.4 Energy restriction reduced serum BDNF in women with low response

To delve deeper into the relationship between serum BDNF levels and an energy-restricted diet, we categorized participants into high and low response groups based on the magnitude of change following the intervention. Subsequently, we analyzed the variance in serum BDNF levels between these groups, while also stratifying by gender to mitigate any gender-related influences on the findings.

Following 4 weeks of this intervention, 30 participants (61.2%) were classified as high responders, while 19 (38.8%) were deemed low responders. Among women, significant differences were observed in BW, BMI, PBF, AC, and VFA (20 high responders and 12 low responders). Among men, the change values for BW, BMI, PBF, BFM, AC, and WHR exhibited significant differences between the high and low response groups (10 high responders and 7 low responders). Consistent with the previous results, BDNF levels were significantly higher in men (*n* = 17) than in women (*n* = 32) before and after an energy restricted diet (18,847.0 ± 5,182.9 pg/ml vs. 15,157.3 ± 5,082.3 pg/ml, *P* = 0.02). However, a noteworthy disparity in BDNF levels was observed between the high and low response groups solely among females (*P* = 0.032), with no such contrast detected among males (*P* = 0.700) (refer to Table 3).

**TABLE 3:** Changes in anthropometric and cardiometabolic parameters stratified according to the weight loss response to the diet intervention (5% weight loss from baseline weight) (*n* = 49).

	Women (*n* = 32)			Men (*n* = 17)		
Variables	High responders	Low responders	*P*	High responders	Low responders	*P*
	***n* = 20**	***n* = 12**		***n* = 10**	***n* = 7**	
ΔBW (kg)	4.3 ± 0.7	2.2 ± 1.1	**<0**.**001**	7.2 ± 2.0	3.0 ± 1.1	**<0**.**001**
ΔBMI (kg/m^2^)	1.6 ± 0.3	0.8 ± 0.5	**<0**.**001**	2.3 ± 0.8	1.2 ± 0.4	**0.002**
ΔSBP (mmHg)	9.5 ± 13.4	6.5 ± 9.9	0.515	7.8 ± 13.8	7.3 ± 15.1	0.943
ΔDBP (mmHg)	4.2 ± 8.2	−1.8 ± 12.6	0.117	5.4 ± 7.5	4.3 ± 10.8	0.804
ΔPR (beat/min)	4.7 ± 6.4	5.0 ± 7.7	0.89	10.2 ± 12.1	9.4 ± 11.5	0.897
ΔFPG (mmol/L)	0.27 ± 0.51	0.12 ± 0.5	0.432	0.82 ± 1.37	0.30 ± 0.83	0.387
ΔTG (mmol/L)	0.62 ± 0.77	0.22 ± 0.39	0.109	2.07 ± 3.38	0.11 ± 0.50	0.153
ΔTC (mmol/L)	0.42 ± 0.52	0.52 ± 0.51	0.602	0.46 ± 0.95	0.08 ± 0.47	0.346
ΔLDL-C (mmol/L)	0.27 ± 0.34	0.37 ± 0.35	0.446	−0.13 ± 0.30	−0.05 ± 0.46	0.705
ΔHDL-C (mmol/L)	0.04 ± 0.17	0.04 ± 0.16	0.983	−0.04 ± 0.11	−0.02 ± 0.16	0.733
ΔBFM (kg)	3.7 ± 1.0	2.3 ± 1.1	**0.001**	5.3 ± 1.6	3.0 ± 1.6	**0.010**
ΔPBF (%)	3.1 ± 1.3	2.2 ± 1.3	0.082	4.0 ± 1.6	2.0 ± 1.1	**0.017**
ΔAC (cm)	5.7 ± 1.4	3.6 ± 1.9	**0.004**	8.7 ± 2.3	4.5 ± 2.4	**0.003**
ΔWHR	0.04 ± 0.02	0.03 ± 0.02	0.189	0.05 ± 0.02	0.03 ± 0.02	**0.037**
ΔVFA (cm^2^)	24.5 ± 7.1	16.3 ± 7.7	**0.004**	26.0 ± 7.3	18.0 ± 10.3	0.077
ΔBDNF (pg/ml)	1,397.16 ± 3,957.76	4,581.17 ± 3,707.34	**0.032**	3,843.53 ± 5,722.08	4,838.13 ± 4,084.24	0.700

Δ represents the value at baseline minus the value after 4 weeks of intervention. *P* indicates the significance analyzed by Student’s *t*-test. Bold indicates *P* values <0.05. BW, body weight; BMI, body mass index; SBP, systolic blood pressure; DBP, diastolic blood pressure; PR, pulse rate; FPG, fasting plasma glucose; TG, triglycerides; TC, total cholesterol; LDL-C, low-density lipoprotein cholesterol; HDL-C, high-density lipoprotein cholesterol; BFM, body fat mass; PBF, percent body fat; AC, abdomen circumference; WHR, waist-to-hip ratio; VFA, visceral fat area; BDNF, brain-derived neurotrophic factor.

**FIGURE 3:**
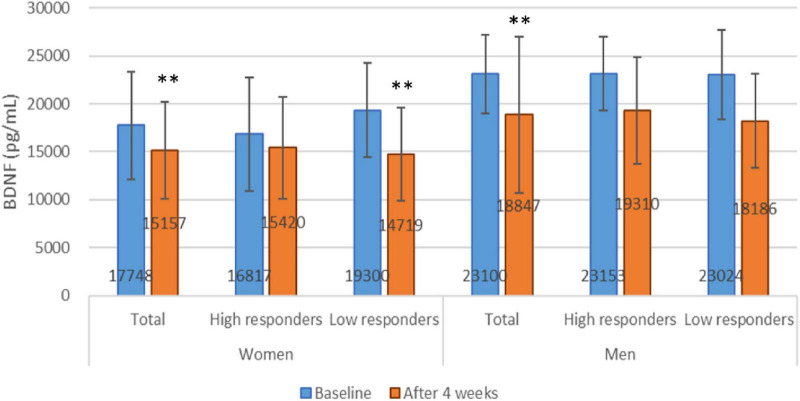
Brain-derived neurotrophic factor levels at baseline and after 4 weeks energy restricted diets. **P < 0.01 compared between baseline and after energy restriction by paired *t*-test.

To further investigate the effects of an energy-restricted diet on BDNF, we conducted a subgroup analysis to examine changes in BDNF levels before and after the diet in high and low response groups of both women and men. In women, BDNF levels were 16,817 ± 5,922.8 and 19,300.4 ± 4,911.4 before, and 15,420.1 ± 5,325.2 and 14,719.3 ± 845.3 after the energy-restricted diet in the high and low response groups, respectively. In men, BDNF levels were 23,153.1 ± 3,873.2 and 23,024.3 ± 4,684.9 before, and 19,309.6 ± 5,577.7 and 18,186.2 ± 4,909.3 after the diet in the high and low response groups, respectively. No significant differences in BDNF levels were observed between the high and low response groups in either women or men before or after the diet. A correlation was found between BDNF levels before and after the diet in the women’s high and low response groups, with correlation coefficients of 0.757 (*P* < 0.001) and 0.711 (*P* = 0.009), respectively. However, paired tests showed that only the low response group had a significant change in BDNF levels before and after the diet (*P* = 0.001), while no significant difference was observed in the high response group (*P* = 0.131). In men, a significant difference was observed in the low response group before and after the diet (*P* = 0.02), though no association was found (correlation coefficients = 0.638, *P* = 0.123). These findings suggest that an energy-restricted diet is associated with decreased BDNF levels in the female low response group (refer to Figure 3).

## 4 Discussion

Our study revealed a significant elevation of BDNF levels in the serum of overweight and obese individuals, with a notable increase observed in men within the Chinese population. Furthermore, the implementation of meal replacements and intermittent fasting resulted in decreased serum BDNF levels, accompanied by reductions in BW. In women, failure to lose weight through an energy restricted diet may be associated with a decrease in BDNF.

The correlation between blood BDNF levels and obesity is a topic of ongoing debate in the literature. Some studies have reported decreased plasma BDNF levels in obese patients with severe metabolic syndrome (20), while others have found elevated serum levels of BDNF in newly diagnosed women with type 2 diabetes (21). Additionally, research has shown significantly lower plasma BDNF levels in obese patients compared to controls (22). However, a meta-analysis involving 307 obese patients and 236 normal controls indicated that BDNF levels in obese patients were similar to those in controls (23).

The apparent inconsistency observed above could stem from various factors impacting serum BDNF levels. Once BDNF enters the bloodstream, approximately 90% of it becomes sequestered in platelets. Research indicates that factors such as age, weight, sex, and menstrual cycle can exert influence over the levels of BDNF stored in platelets as well as plasma BDNF in healthy adults (24). Research has shown that fluctuations in ovarian hormones, such as estradiol, during the menstrual cycle can significantly impact BDNF levels (24, 25). Furthermore, the transition into menopause, characterized by significant hormonal fluctuations has been associated with changes of BDNF levels (26). In the context of our current investigation, the distribution of age across the normal, overweight, and obese groups was comparable. BDNF levels displayed significant variability in women, which is related to the factor of unaccounted menstrual cycles. However, BDNF levels in men showed significantly higher than in women. Physical activity is another confounding variables for BDNF levels. Many studies demonstrated that physical exercise, either moderate-intensity exercises or acute aerobic exercise, can effectively increase peripheral BDNF levels (27–29). In this study, all participants engaged in appropriate exercise. However, neither the types nor the duration of physical activity were documented or regulated.

Second, the debate on the association between obesity and BDNF levels may be related to genetic polymorphisms in BDNF in different populations. It is known that certain polymorphisms may serve as genetic determinants of obesity (30, 31). A comprehensive study analyzing the association between BMI and approximately 2.8 million SNPs in over 123,865 individuals identified a significant correlation between obesity and a 32-locus, notably implicating rs10767664 proximal to the BDNF gene (32). Significant association between the presence of rs6265 in BDNF (Val66Met SNP) and obesity in ethnically homogenous groups of healthy Caucasian children and adolescents (33), as well as Chinese children (34, 35). This polymorphism strongly influences reduced circulating leptin levels, heightened hypothalamic BDNF expression, and consequently, increased food intake and overweight, particularly evident in BDNF Met/Met mice (36). Additionally, the BDNF-rs7934165-AA genotype has been linked to a higher waist-to-height ratio, suggesting its potential utility as a biomarker for central obesity and cardiometabolic risk (37).

Gender disparities in the association of BDNF rs6265 genotype with obesity have been observed. Ma et al. (38) demonstrated that men with the GG genotype exhibit higher BMI, waist circumference, hip circumference, and weight compared to GA or AA carriers. Conversely, women with the GG genotype displayed significantly lower BMI, buttocks circumference, and BW than GA or AA carriers (38). BDNF polymorphisms might influence serum concentration and plasma BDNF levels further correlate with obesity and the BDNF Val66Met SNP (22). Genetic polymorphisms may be a potential factor for the differences in serum BDNF levels found in this study among normal weight, overweight and obese men only. Our study analyzed the rs6265 polymorphism and found no association with serum BDNF content or with weight changes induced by meal-replacement-assisted intermittent fasting (data not shown). The lack of significant findings may be attributed to the limited sample size. Other BDNF polymorphisms were not examined.

Third, several other factors, such as subject recruitment methods, sampling techniques, and procedures for handling and storing samples, can introduce deviations that may impact the accuracy of BDNF measurements (23). Notably, serum BDNF, in contrast to plasma, exhibits greater stability and can be stored for prolonged periods. However, it is susceptible to variations induced by coagulation temperature and duration (39). In our study, serum samples were uniformly left to stand at room temperature for 3 h before being centrifuged at 1200 *g* for 10 min. These differing measurement parameters could account for disparities between our study and others in the field.

Various forms of energy-restricted diets, such as calorie restriction, alternate-day fasting, time-limited eating, and Ramadan with intermittent fasting, have demonstrated efficacy in reducing weight among obese individuals (18, 40, 41). In this study, we explored the impact of a calorie restriction-assisted intermittent fasting regimen on obesity. Our findings reveal that 62.5% of women and 58.8% of men exhibited a high responsiveness to this dietary pattern.

Dietary interventions and physical exercises have been shown to influence BDNF levels in the body. Consistent with our findings, Glud et al. (17) reported a 22% reduction in circulating BDNF levels in both men and women following exercise alone. Additionally, Merhi et al. (42) demonstrated approximately a 50% reduction in circulating BDNF levels and a 12.6% decrease in BMI 3 months post-bariatric surgery in 18 morbidly obese women. However, Harvie et al. (43) reported no significant change in serum BDNF levels among overweight women following intermittent or continuous energy restriction. This finding may be attributed to the sustained 25% energy restriction intervention, resulting in only a 3.5% weight loss over 6 months. However, our study implemented meal replacements and intermittent fasting over 1 month, resulting in a 5% weight loss (43). In contrast to this study, where BDNF levels significantly decreased in both men and women before and after energy restriction, significant reductions of 29.9% and 32.5% in BDNF were observed in women, but not in men, during the low-energy diet alone or when combined with exercise (17). This is likely due to the relatively low baseline levels of BDNF in men prior to the intervention in that study (17).

In the subgroup analysis, it was found that only in women did the low responder group exhibit a greater reduction in BDNF compared to the high responder group, whereas no differences in BDNF changes were observed between the high and low responder groups in men. And it was intriguing that the energy-restricted diet was linked to reduced BDNF levels exclusively in the female low response group. The sex-specific changes in BDNF due to energy-restricted diets may be linked to gender differences in energy metabolism, including glucose response to low-energy diets or exercise interventions (44). The results hint that maintaining relatively stable BDNF levels is crucial for successful weight loss through energy restriction diets in women. Approaches to boost BDNF, such as incorporating BDNF-enhancing foods or exercise (45), could potentially be integrated into the intervention.

The relationship between energy-restricted diets, weight loss, and neuroendocrine mechanisms, such as the regulation of appetite, is a complex interplay involving various hormones and signaling pathways. BDNF, one of the key players in this process, is known to be involved in energy homeostasis and appetite regulation (7, 8). Higher BDNF in overweight/obese individuals and decreased BDNF following energy-restricted diets and subsequent weight loss may suggest alterations in neuroendocrine signaling pathways that influence appetite and energy balance.

Limitations of this study include an insufficient sample size, particularly a notable shortage of male participants during the energy restriction intervention phase, which limited our ability to assess its impact on BDNF levels. Confounding variables, including physical activity, menopausal status, and menstrual cycle, were neither recorded, analyzed, nor controlled. Additionally, the relatively short duration of the intervention precluded an examination of the long-term effects of energy-restricted diets on obesity and changes in blood BDNF levels. Whether BDNF levels return to normal following energy-restricted diets remains unclear. This study focused solely on the Chinese population, and its findings, along with those from other populations, warrant further investigation.

## 5 Conclusion

Serum BDNF levels were found to be elevated in overweight and obese adult man, and an energy-restricted diet led to a reduction in serum BDNF in Chinese population. Among women, the inability to achieve weight loss with an energy-restricted diet may be linked to decreased BDNF levels. This work offers valuable insights into the relationship between blood BDNF levels, obesity, and energy restriction in a real-world context. It also suggests that maintaining relatively stable BDNF levels, for example, by incorporating methods such as exercise, is essential for successful weight loss through energy-restricted diets. Future clinical studies with a larger sample size and a more comprehensive design, taking multiple factors such as menstrual cycle, menopausal status and physical activity into account, are warranted.

## Data Availability

The raw data supporting the conclusions of this article will be made available by the authors, without undue reservation.

## References

[1] National Institutes of Health. *Health Risks of Overweight and Obesity.* (2023). Available online at: https://www.niddk.nih.gov/health-information/weight-management/health-risks-overweight (accessed June 7, 2024).

[2] GBD 2019 Risk Factors Collaborators. Global burden of 87 risk factors in 204 countries and territories, 1990-2019: A systematic analysis for the global burden of disease study 2019. *Lancet.* (2020) 396:1223–49. 10.1016/S0140-6736(20)30752-2 33069327 PMC7566194

[3] Okunogbe A Nugent R Spencer G Powis J Ralston J Wilding J. Economic impacts of overweight and obesity: Current and future estimates for 161 countries. *BMJ Glob Health.* (2022) 7:e009773. 10.1136/bmjgh-2022-009773 36130777 PMC9494015

[4] Catenacci V Pan Z Ostendorf D Brannon S Gozansky W Mattson M A randomized pilot study comparing zero-calorie alternate-day fasting to daily caloric restriction in adults with obesity. *Obesity (Silver Spring).* (2016) 24:1874–83. 10.1002/oby.21581 27569118 PMC5042570

[5] Ozek C Zimmer D De Jonghe B Kalb R Bence K. Ablation of intact hypothalamic and/or hindbrain TrkB signaling leads to perturbations in energy balance. *Mol Metab.* (2015) 4:867–80. 10.1016/j.molmet.2015.08.002 26629410 PMC4632115

[6] Javed S Chang Y Cho Y Lee Y Chang H Haque M Smith-Magenis syndrome protein RAI1 regulates body weight homeostasis through hypothalamic BDNF-producing neurons and neurotrophin downstream signalling. *Elife.* (2023) 12:R90333. 10.7554/eLife.90333 37956053 PMC10642964

[7] Marosi K Mattson MP. BDNF mediates adaptive brain and body responses to energetic challenges. *Trends Endocrinol Metab.* (2014) 25:89–98. 10.1016/j.tem.2013.10.006 24361004 PMC3915771

[8] Wang P Loh K Wu M Morgan D Schneeberger M Yu X A leptin-BDNF pathway regulating sympathetic innervation of adipose tissue. *Nature.* (2020) 583:839–44. 10.1038/s41586-020-2527-y 32699414

[9] Pius-Sadowska E Machaliński B. BDNF - A key player in cardiovascular system. *J Mol Cell Cardiol.* (2017) 110:54–60. 10.1016/j.yjmcc.2017.07.007 28736262

[10] Rentería I García-Suárez P Fry A Moncada-Jiménez J Machado-Parra J Antunes B The molecular effects of BDNF synthesis on skeletal muscle: A mini-review. *Front Physiol.* (2022) 13:934714. 10.3389/fphys.2022.934714 35874524 PMC9306488

[11] Rios M. BDNF and the central control of feeding: Accidental bystander or essential player? *Trends Neurosci.* (2013) 36:83–90. 10.1016/j.tins.2012.12.009 23333344 PMC3568936

[12] Fulgenzi G Hong Z Tomassoni-Ardori F Barella L Becker J Barrick C Novel metabolic role for BDNF in pancreatic β-cell insulin secretion. *Nat Commun.* (2020) 11:1950. 10.1038/s41467-020-15833-5 32327658 PMC7181656

[13] Gholami F Mesrabadi J Iranpour M Donyaei A. Exercise training alters resting brain-derived neurotrophic factor concentration in older adults: A systematic review with meta-analysis of randomized-controlled trials. *Exp Gerontol.* (2025) 199:112658. 10.1016/j.exger.2024.112658 39674562

[14] Behrad S Dezfuli S Yazdani R Hayati S Shanjani S. The effect of physical exercise on circulating neurotrophic factors in healthy aged subjects: A meta-analysis and meta-regression. *Exp Gerontol.* (2024) 196:112579. 10.1016/j.exger.2024.112579 39260585

[15] Cheng M Cong J Wu Y Xie J Wang S Zhao Y Chronic swimming exercise ameliorates low-soybean-oil diet-induced spatial memory impairment by enhancing BDNF-mediated synaptic potentiation in developing spontaneously hypertensive rats. *Neurochem Res.* (2018) 43:1047–57. 10.1007/s11064-018-2515-x 29574667

[16] Khabour O Alzoubi K Alomari M Alzubi M. Changes in spatial memory and BDNF expression to simultaneous dietary restriction and forced exercise. *Brain Res Bull.* (2013) 90:19–24. 10.1016/j.brainresbull.2012.08.005 23000024

[17] Glud M Christiansen T Larsen L Richelsen B Bruun J. Changes in circulating BDNF in relation to sex, diet, and exercise: A 12-week randomized controlled study in overweight and obese participants. *J Obes.* (2019) 2019:4537274. 10.1155/2019/4537274 31781387 PMC6875316

[18] Alkurd R Mahrous L Zeb F Khan M Alhaj H Khraiwesh H Effect of calorie restriction and intermittent fasting regimens on brain-derived neurotrophic factor levels and cognitive function in humans: A systematic review. *Medicina (Kaunas).* (2024) 60:191. 10.3390/medicina60010191 38276070 PMC10819730

[19] Zhou B. Predictive values of body mass index and waist circumference for risk factors of certain related diseases in Chinese adults–study on optimal cut-off points of body mass index and waist circumference in Chinese adults. *Biomed Environ Sci.* (2002) 15:83–96. 12046553

[20] Chaldakov G Fiore M Stankulov I Hristova M Antonelli A Manni L NGF, BDNF, leptin, and mast cells in human coronary atherosclerosis and metabolic syndrome. *Arch Physiol Biochem.* (2001) 109:357–60. 10.1076/apab.109.4.357.4249 11935372

[21] Suwa M Kishimoto H Nofuji Y Nakano H Sasaki H Radak Z Serum brain-derived neurotrophic factor level is increased and associated with obesity in newly diagnosed female patients with type 2 diabetes mellitus. *Metabolism.* (2006) 55:852–7. 10.1016/j.metabol.2006.02.012 16784955

[22] Zamani M Hosseini S Behrouj H Erfani M Dastghaib S Ahmadi M BDNF Val66Met genetic variation and its plasma level in patients with morbid obesity: A case-control study. *Gene.* (2019) 705:51–4. 10.1016/j.gene.2019.04.045 31004714

[23] Sandrini L Di Minno A Amadio P Ieraci A Tremoli E Barbieri S. Association between obesity and circulating brain-derived neurotrophic factor (BDNF) levels: Systematic review of literature and meta-analysis. *Int J Mol Sci.* (2018) 19:2281. 10.3390/ijms19082281 30081509 PMC6121551

[24] Lommatzsch M Zingler D Schuhbaeck K Schloetcke K Zingler C Schuff-Werner P The impact of age, weight and gender on BDNF levels in human platelets and plasma. *Neurobiol Aging.* (2005) 26:115–23. 10.1016/j.neurobiolaging.2004.03.002 15585351

[25] Oral E Ozcan H Kirkan T Askin S Gulec M Aydin N. Luteal serum BDNF and HSP70 levels in women with premenstrual dysphoric disorder. *Eur Arch Psychiatry Clin Neurosci.* (2013) 263:685–93. 10.1007/s00406-013-0398-z 23455589

[26] Gordon J Eisenlohr-Moul T Rubinow D Schrubbe L Girdler S. Naturally occurring changes in estradiol concentrations in the menopause transition predict morning cortisol and negative mood in perimenopausal depression. *Clin Psychol Sci.* (2016) 4:919–35. 10.1177/2167702616647924 27867758 PMC5113718

[27] Coelho F Gobbi S Andreatto C Corazza D Pedroso R Santos-Galduróz R. Physical exercise modulates peripheral levels of brain-derived neurotrophic factor (BDNF): A systematic review of experimental studies in the elderly. *Arch Gerontol Geriatr.* (2013) 56:10–5. 10.1016/j.archger.2012.06.003 22749404

[28] Máderová D Krumpolec P Slobodová L Schön M Tirpáková V Kovaničová Z Acute and regular exercise distinctly modulate serum, plasma and skeletal muscle BDNF in the elderly. *Neuropeptides.* (2019) 78:101961. 10.1016/j.npep.2019.101961 31506171

[29] Ribeiro D Petrigna L Pereira F Muscella A Bianco A Tavares P. The impact of physical exercise on the circulating levels of BDNF and NT 4/5: A review. *Int J Mol Sci.* (2021) 22:8814. 10.3390/ijms22168814 34445512 PMC8396229

[30] Xu B Xie X. Neurotrophic factor control of satiety and body weight. *Nat Rev Neurosci.* (2016) 17:282–92. 10.1038/nrn.2016.24 27052383 PMC4898883

[31] Marcos-Pasero H Aguilar-Aguilar E Ikonomopoulou M Loria-Kohen V. BDNF Gene as a precision skill of obesity management. *Adv Exp Med Biol.* (2021) 1331:233–48. 10.1007/978-3-030-74046-7_15 34453302

[32] Speliotes E Willer C Berndt S Monda K Thorleifsson G Jackson A Association analyses of 249,796 individuals reveal 18 new loci associated with body mass index. *Nat Genet.* (2010) 42:937–48. 10.1038/ng.686 20935630 PMC3014648

[33] Skledar M Nikolac M Dodig-Curkovic K Curkovic M Borovecki F Pivac N. Association between brain-derived neurotrophic factor Val66Met and obesity in children and adolescents. *Prog Neuropsychopharmacol Biol Psychiatry.* (2012) 36:136–40. 10.1016/j.pnpbp.2011.08.003 21851847

[34] Wu L Xi B Zhang M Shen Y Zhao X Cheng H Associations of six single nucleotide polymorphisms in obesity-related genes with BMI and risk of obesity in Chinese children. *Diabetes.* (2010) 59:3085–9. 10.2337/db10-0273 20843981 PMC2992769

[35] Wu L Gao L Zhao X Zhang M Wu J Mi J. Associations of two obesity-related single-nucleotide polymorphisms with adiponectin in Chinese children. *Int J Endocrinol.* (2017) 2017:6437542. 10.1155/2017/6437542 28396685 PMC5370521

[36] Ieraci A Barbieri S Macchi C Amadio P Sandrini L Magni P BDNF Val66Met polymorphism alters food intake and hypothalamic BDNF expression in mice. *J Cell Physiol.* (2020) 235:9667–75. 10.1002/jcp.29778 32430940

[37] Guerrero-Contreras I Hernández-Tobías E Velázquez-Cruz R Ramírez-López E Campos-Góngora E Jiménez-Salas Z. The BDNF rs7934165 polymorphism is a biomarker of central obesity and cardiometabolic risk in Mexican women. *Eur Rev Med Pharmacol Sci.* (2021) 25:5463–73. 10.26355/eurrev_202109_26655 34533795

[38] Ma X Qiu W Smith C Parnell L Jiang Z Ordovas J Association between BDNF rs6265 and obesity in the Boston puerto rican health study. *J Obes.* (2012) 2012:102942. 10.1155/2012/102942 23326649 PMC3543800

[39] Amadio P Sandrini L Ieraci A Tremoli E Barbieri S. Effect of clotting duration and temperature on BDNF measurement in human serum. *Int J Mol Sci.* (2017) 18:1987. 10.3390/ijms18091987 28914800 PMC5618636

[40] Jamshed H Steger F Bryan D Richman J Warriner A Hanick C Effectiveness of early time-restricted eating for weight loss, fat loss, and cardiometabolic health in adults with obesity: A randomized clinical trial. *JAMA Intern Med.* (2022) 182:953–62. 10.1001/jamainternmed.2022.3050 35939311 PMC9361187

[41] Pavlou V Cienfuegos S Lin S Ezpeleta M Ready K Corapi S Effect of time-restricted eating on weight loss in adults with type 2 diabetes: A randomized clinical trial. *JAMA Netw Open.* (2023) 6:e2339337. 10.1001/jamanetworkopen.2023.39337 37889487 PMC10611992

[42] Merhi Z Minkoff H Lambert-Messerlian G Macura J Feldman J Seifer D. Plasma brain-derived neurotrophic factor in women after bariatric surgery: A pilot study. *Fertil Steril.* (2009) 91:1544–8. 10.1016/j.fertnstert.2008.09.032 18950757

[43] Harvie M Pegington M Mattson M Frystyk J Dillon B Evans G The effects of intermittent or continuous energy restriction on weight loss and metabolic disease risk markers: A randomized trial in young overweight women. *Int J Obes (Lond).* (2011) 35:714–27. 10.1038/ijo.2010.171 20921964 PMC3017674

[44] Mauvais-Jarvis F. Sex differences in energy metabolism: Natural selection, mechanisms and consequences. *Nat Rev Nephrol.* (2024) 20:56–69. 10.1038/s41581-023-00781-2 37923858

[45] Xue B Waseem S Zhu Z Alshahrani M Nazam N Anjum F Brain-derived neurotrophic factor: A connecting link between nutrition, lifestyle, and Alzheimer’s disease. *Front Neurosci.* (2022) 16:925991. 10.3389/fnins.2022.925991 35692417 PMC9177140

